# Reproductive Disorders and Leptospirosis: A Case Study in a Mixed-Species Farm (Cattle and Swine)

**DOI:** 10.3390/vetsci4040064

**Published:** 2017-12-01

**Authors:** Marcella Mori, Raïssa Bakinahe, Philippe Vannoorenberghe, Jo Maris, Ellen de Jong, Marylène Tignon, Martine Marin, Damien Desqueper, David Fretin, Isabelle Behaeghel

**Affiliations:** 1 Bacterial Zoonoses of Livestock, Operational Directorate Bacterial Diseases, Veterinary and Agrochemical Research Centre, CODA-CERVA, 1180 Brussels, Belgium; Raissa.Bakinahe@coda-cerva.be (R.B.); Philippe.Vannoorenberghe@coda-cerva.be (P.V.); Martine.Marin@coda-cerva.be (M.M.); Damien.Desqueper@coda-cerva.be (D.D.); David.Fretin@coda-cerva.be (D.F.); 2 Dierengezondheidszorg Vlaanderen, DGZ, 8820 Torhout, Belgium; Jo.Maris@boehringer-ingelheim.com (J.M.); Ellen.deJong@dgz.be (E.d.J.); 3 Virology, Operational Directorate Enzotic and (re)emerging diseases, Veterinary and Agrochemical Research Centre, CODA-CERVA, 1180 Brussels, Belgium; Marylene.Tignon@coda-cerva.be; 4 Epidemiology, Operational Directorate Interaction and surveillance, Veterinary and Agrochemical Research Centre, CODA-CERVA, 1180 Brussels, Belgium; Isabelle.Behaeghel@coda-cerva.be

**Keywords:** reproductive problems, spontaneous abortion, diagnosis of leptospirosis, diagnostic cut-off, PCR

## Abstract

Animal leptospirosis, exempt in rodents, manifests as peculiar biology where the animal can function, simultaneously or not, as a susceptible host or reservoir. In the first case, clinical symptoms are likely. In the second case, infection is subclinical and manifestations are mild or absent. Mild clinical symptoms encompass reproductive failure in production animals for host-adapted *Leptospira* sp. serovars. This work presents a study on *Leptospira* sp. infection in a mixed-species (bovine and swine) farm with documented reproductive disorders in the cattle unit. A long calving interval (above 450 days) was the hallmark observed in cows. Some cows (2/26 tested) presented a high titre of antibodies against *Leptospira* sp. serogroup Sejroe, but the overall within-herd prevalence was low (11.5% and 7.7% for cut-off titres of 1:30 and 1:100, respectively). The in-herd prevalence of leptospirosis in the sow unit (determined for 113/140 animals) was high when using a lowered cut-off threshold (32.7% vs. 1.8% for cut-off titre of 1:30 and 1:100, respectively). In this unit, the most prevalent serogroup was Autumnalis. The final diagnostic confirmation of *Leptospira* sp. maintenance within the farm was obtained through detection by PCR of *Leptospira* sp. DNA in an aborted swine litter. Despite the fact that a common causative infective agent was diagnosed in both species, the direct link between the two animal units was not found. Factors such as drinking from the same water source and the use of manure prepared with the swine slurry might raise suspicion of a possible cross-contamination between the two units. In conclusion, this work suggests that leptospirosis be included in the differential diagnosis of reproductive disorders and spontaneous abortions in production animals and provides data that justify the use of a lowered threshold cut-off for herd diagnosis.

## 1. Introduction

Reproductive disorders comprise a plethora of clinical manifestations spanning from infertility to spontaneous abortion and can be associated with simple or more complex dynamics, implicating, among others, infective agents. Leptospiral infection has been reported as one cause of reproductive disorders in production animals [1]. Leptospirosis is a zoonotic disease with global distribution, caused by spirochetes belonging to the genus Leptospira. The infection is transmitted from an infected host to the recipient (animal or human) by direct or indirect contact with water, soil or another milieu contaminated with infected urine. The epidemiology, the wide range of susceptible and reservoir hosts, an intricate ecology and limitation in cultivating the bacterium make this disease sometimes difficult to diagnose in production animals, particularly in its subclinical forms. The diagnostic tests most widely used in this context are indirect serology and/or direct confirmation of bacterial presence by isolation or PCR, in the early phase of acute infection [2] or chronic animal shedders [3,4]. Serology is obtained by titration of *Leptospira* sp. antibodies in infected animals with the microscopic agglutination test (MAT). The World Organisation for Animal Health (OIE) manual recommends that this test be performed with live *Leptospira* sp. as antigen [5]. The specificity of this test is high but alone it is not sufficient to provide a definitive leptospirosis diagnosis, unless a four-fold rise of titre in convalescent sera is demonstrated [5]. On the other hand, serological diagnosis in production animals is more complex, because animals can be maintenance hosts and the serological status of infection can be associated with low or absent levels of *Leptospira* sp. antibodies [6].

In the absence of a perfectly satisfactory diagnostic test, MAT and PCR are used complementarily. For the MAT, the standard protocol [5] indicates the use in routine diagnosis of a cut-off threshold of 1:100 irrespective of the diagnosed species. Lowering the threshold can be acceptable under specific circumstances such as in serosurveillance studies [7]. This report presents a case of reproductive disorders in a cattle herd associated with high serology titre of *Leptospira* sp. serogroup Sejroe antibodies but no direct detection of the pathogen. In the second herd present on this mixed-species farm, the swine unit, antibodies against *Leptospira* sp. were at low titre and would have been unnoticed at 1:100 threshold dilutions. In this herd, the presence of *Leptospira* sp. was finally confirmed by indirect and direct diagnosis of the pathogen in two consecutive aborted litters.

## 2. Materials and Methods

### 2.1. Study Design

#### 2.1.1. Cattle: Reproductive Performance

Reproductive performance of the cattle unit was described by calculating the calving interval and the conception rate, defined as the percentage of inseminations per cow necessary to result in pregnancy, and by monitoring the cycling of the cows. Management regarding reproduction was also assessed.

#### 2.1.2. Cattle Sample Size

The calculation of the total number of cows to be sampled was based on an expected prevalence of 50% (considering no a priori knowledge of the prevalence), an accepted error of 10% and a 90% confidence level. Since the population size was 107 cows, a total sample size of 31 animals was originally determined.

#### 2.1.3. Pigs

The calculation of the total number of sows to be sampled was based on an expected prevalence of 50% (considering no a priori knowledge of the prevalence), an accepted error of 5% and a 95% confidence level. Since the population size was 140 sows, a total sample size of 103 animals was determined. No detailed data regarding reproductive performance of the sows were available.

### 2.2. Sample Collection

Blood samples were collected in serum tubes, centrifuged and the serum stored at −20 °C until analysis. Urine samples were taken by catheterization of three cows, two of which tested positive by the microagglutination test. The aborted piglets were also preserved in cooled conditions and organ tissues were sampled and analysed between one and three days after spontaneous abortion had occurred.

### 2.3. Strain and Culture Conditions

The Leptospira strains were maintained in a liquid Ellinghausen–McCullough–Johnson–Harris (EMJH) medium supplemented with 0.2% *w*/*v* yeast extract (both from Difco, Becton Dickinson, Benelux nv. Dorp 86, 9320 Erembodege, Belgium.) and 10% foetal calf serum (PAA laboratories GmbH, A&E Scientific Rue de Lekernay, 7850 Enghien, Belgium.). Cultures were grown at 29 °C and inoculated weekly by 1:50 dilution. Strains are controlled every six months upon a panel of positive serovar-specific antisera (KIT, Amsterdam, The Netherlands).

### 2.4. Serum Microscopic Agglutination Test (MAT)

The MAT was performed using a panel of 12 live cultured *Leptospira* strains comprising the following serogroups, with related serovars in brackets, Grippotyphosa (grippotyphosa), Canicola (canicola), Pomona (pomona), Ballum (castellonis), Icterohaemorrhagiae (icterohaemorrhagiae), Javanica (poi), Australis (muenchen), Autumnalis (autumnalis), Bataviae (bataviae), Pyrogenes (pyrogenes), Tarassovi (tarassovi), Sejroe (Hardjo type prajitno) (MAT12) (Supplementary Materials Table S1). The procedure was that recommended by the OIE manual [5]. Briefly, sera were diluted in PBS (Thermo Fisher Diagnostics nv, Guldensporenpark 26, 9820 Merelbeke, Belgium) and live strains to obtain the desired threshold dilution and incubated for 1 h at 39 °C. Endpoint titres were determined starting from an initial dilution of 1:10 and using a three-fold dilution until the last condition showing 50% agglutination was observed.

### 2.5. Diagnostic Real-Time PCR for Leptospirosis

For nucleic acid extraction, tissue samples were smashed in a minimum of 250 µL of physiological water and 200 µL of this homogenate were mixed with 235 µL lysis-binding solution (MagMax, Applied Biosystems, Life Technologies bv, Gent, Belgium) supplemented with lysozyme (Sigma-Aldrich, Brusselsesteenweg 288, 3090 Overijse, Belgium) at a final concentration of 1 mg/mL. After incubation for one hour at 37 °C, samples were processed for extraction as defined by the manufacturer (MagMax nucleic acid extraction kit, Applied Biosystems). For the amplification of the Lipl32 gene, primers, probe and thermal conditions were as previously described [8]: the set included LipL32-45F (5′-AAGCATTACCGCTTGTGGTG-3′), LipL32-286R (5′-GAACTCCCATTT CAGCGATT-3′) and the probe LipL32-189P (FAM-5′-AAAGCCAGGACAAGCGCCG-3′- Quencher BHQ1) (Eurogentec, Rue du Bois Saint-Jean 5, 4102 Seraing, Belgium). The reaction mix consisted of 2× absolute mix (Applied Biosystem), 540 nM each of forward and reverse primers and 160 nM of TaqMan probe. Thermal conditions were 2 min at 50 °C, 15 min at 95 °C and subsequent 45 cycles of amplification (15 s at: 95 °C, 1 min at 58 °C, 30 s at 72 °C).

### 2.6. Diagnostic Real-Time PCR for Hepatitis E Virus (HEV) and Porcine Reproductive and Respiratory Syndrome Virus (PRRSV)

Viral RNA was extracted using Viral RNA kit (Qiagen, Quellinstraat49, 2018 Antwerp, Belgium) and used for cDNA synthesis with random hexamers (Roche Life Science, Schaarbeeklei 198, 1800 Vilvoorde, Belgium) and M-MLV Reverse transcriptase polymerase (Invitrogen, 9820 Merelbeke, Belgium). The presence of an HEV-specific sequence was investigated in the samples by real-time PCR with the primers and probe described by Jothikumar et al. (2006) [9]. The PRRS-specific sequences were detected with a SYBR Green PCR assay using primers designed by Martinez et al. (2008) [10]. The two real-time PCR assays were performed on a LightCyler 480 (Roche Life Science) using adapted master mixes (LightCycler FastStart DNA Taqman probe Master Mix and FastStart DNA Master SYBR Green I) from Roche Life Science and according to the manufacturer’s recommendations.

## 3. Results

### 3.1. Description of the Farm at the Time of Study

In the late fall of 2012, a case of a mixed-units farm presented at the National Reference Laboratory for leptospirosis with history of poor fertility performance among the cattle unit. The farm consisted of two different species units with the following characteristics:

- Cattle herd: 107 animals with age and sex distribution as indicated in Table 1, Holstein–Friesian breed. On average, 50 lactating cows;
- Sow unit: 140 animals. Record-keeping of the sanitary parameters in this herd was not provided.

The two units were not in contact due to the intensive management of the farm, with the exception that the two units were drinking from the same water source (wellbore) and manure was prepared from the swine slurry. The latter was used as fertilizer for the cow’s grazing area. The farm presented clear infestation with common rats (*Rattus norvegicus*), particularly within the swine unit.

The veterinary parameters of the cattle herd were well documented. Reproductive problems were mainly observed in the between-calving intervals, which were 450–500 days. The conception rate was low (3.67), as well as the success rate of offspring (2/10). Artificial insemination has been performed by the owner for the past 18 years and no particular spontaneous abortion problems were recorded between 2011 and 2012. Nevertheless, heat detection and the insemination protocol were reviewed and the nitrogen tank was checked. No abnormalities were observed. The cattle herd was Bovine Viral Diarrhoea Virus (BVDV) (2×/year) and Infectious Bovine Rhinotracheitis Virus (IBRV) (1×/year) vaccinated, and prevention of young cattle with antiparasitic injection before and after the grazing season occurred. Clinical signs of paratuberculosis were absent and no spontaneous abortions were present that would make us suspect Neospora infection. Metabolic parameters (hypocalcaemia, LDL, laminitis and sleeping milk disease) were within the normal reference level. The animals were of general good size, attentive and active, with optimal skin conditions, mammary gland status within reference values (rarely mastitis). An extensive laboratory follow-up was performed including BVDV antigens and salmonella in tank milk, selenium and iodine, IBRV IgE and *Leptospira* antibodies. Of the above analyses, all were uninformative, with the exception of two out of six tested animals presenting antibodies against *Leptospira* sp. serogroup Serjoe at threshold dilutions of 1:300 and 1:500, respectively.

### 3.2. Seroprevalence and Bacteriology of Leptospira within the Cattle Population

The in-herd prevalence in cattle was next evaluated by MAT12 in 26 animals that were actually sampled, eight of them sampled twice at an interval of 4–8 weeks. The prevalence was estimated at 15.4% (95% CI: 3.3–27.5), 11.5% (95% CI: 0.8–22.2), and 7.7% (95% CI: 0.0–16.6) for cut-off titre of 1:10, 1:30 and 1:100, respectively. Represented serogroups were Sejroe (2), Australis (2), Javanica and Ballum (one animal with mixed serogroups). Only cows older than two years were affected. Twenty-one blood samples were additionally tested for the presence of *Leptospira* DNA and were negative. Three urine samples, two collected from the two animals presenting antibody titres ≥1:300 against serogroup Sejroe, were deemed negative by diagnostic PCR.

### 3.3. Seroprevalence of Leptospira Antibodies in Sows

**Figure 1:**
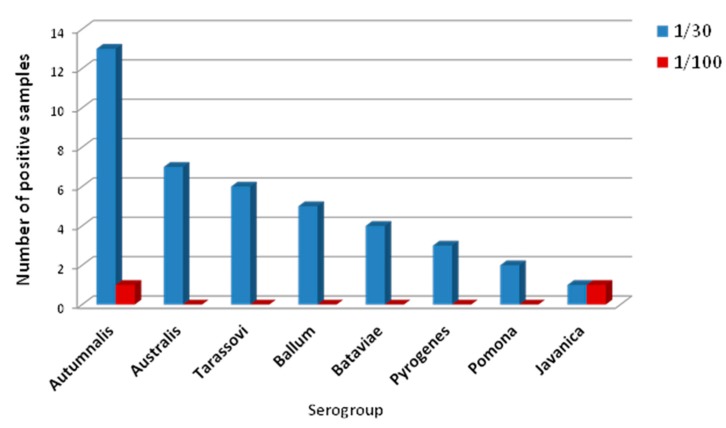
Distribution of *Leptospira* serogroups in the swine unit in 113 animals. The chart shows the results among 37 sows positive for MAT with a cut-off threshold of 1/30. The bars indicate the distribution of the *Leptospira* serogroups, considering the most prevalent serogroup.

The estimated in-herd seroprevalence for leptospirosis was calculated on 113 sows that were sampled and corresponded to 84.1% (95% CI: 84.4–89.8), 32.7% (95% CI: 28.9–36.5) and 1.8% (95% CI: 0.7–2.8) for cut-off titre of 1:10, 1:30 and 1:100, respectively. The distribution of the serogroups for the 1:30 dilution was Autumnalis (32%), Australis (17%), Tarassovi (15%), Ballum (12%), Bataviae (10%), Pyrogenes (7%), Pomona (5%), and Javanica (2%) (Figure 1). At the suggested diagnostic cut-off dilution of 1:100, all but two sows were negative and none was positive beyond this dilution (Figure 1).

### 3.4. Carriage of Leptospira in Aborted Piglets

Within two months of the beginning of the study, two spontaneous abortion events occurred in the swine herd. The aborted piglets were analysed for the presence of *Leptospira* antibodies in body fluids (pleural liquid collected during autopsy) and DNA in tissues. No particular visual abnormalities were recorded during autopsy. In the first litter, both piglets presented antibodies against *Leptospira* sp. for various serovars, with no presence of DNA in the tissues tested (kidneys and liver). In the second litter, no antibodies were detected. On the other hand, six out of seven piglets demonstrated *Leptospira* DNA in at least one organ (Table 2). The organs most affected were spleen (4/7) and kidneys (3/7). Bacterial load, ascertained by the average Ct value, was higher in the spleen (average of 32.5) (Table 2) than in the other organs tested. The overall tissue samples tested negative for *Chlamydia* spp. and *C. burnetii* DNA. Slurry (two individual samples) and organs (kidneys and liver) of dead rats (three) were analysed for the presence of *Leptospira* DNA and came back negative.

## 4. Discussion

Leptospirosis in cattle and swine might be a cause of reproductive disorders and spontaneous abortions, leading to significant economic losses [11,12,13]. In these production animals, subclinical forms of the disease are common [14] and often go underdiagnosed. A veterinary practitioner might look for leptospirosis only when negative results arise from investigations in other diseases. In this case report, *Leptospira* infection was identified in a mixed-species farm. The likely source of transmission/cross-contamination between the units was waterborne (sharing the same water source) or related to the use of manure prepared with the swine slurry. The reproductive problems were clearly documented in the cattle unit and associated with high calving-to-conception intervals, low conception rate and very low success of offspring. Laboratory testing was negative for various infective agents and biochemical parameters except for *Leptospira* serogroup Sejroe antibodies that were identified in two animals. Additional investigations on a larger number of cows in the unit provided evidence of low in-herd seroprevalence. Cattle are maintenance hosts for the serovar Hardjo (Hardjobovis and/or Hardjoprajitno) both belonging to the Sejroe serogroup, and in these cases the disease course evolves towards chronic infections leading to abortion, stillbirth, premature birth or the birth of weak calves [14,15]. The serological titres in case of maintenance serovars are high at the time of clinical onset (i.e., abortion) in half of the animals having aborted [16]. In the rest of cases and in non-aborting cows presenting a subclinical form of the disease, serology is low with no detectable antibodies [16]. It is therefore not surprising that only two animals were serologically positive in the cattle unit of this case report. The low in-herd prevalence will not necessarily exclude other animals in the unit being infected with no measurable level of antibodies. Molecular diagnosis in blood and urine samples of cows did not contribute to a final diagnosis. On the other hand, bacteraemia is transient [17] and shedding in urine intermittent [18,19]. In the swine unit, the clinical scenario was less documented, albeit irregular cases of spontaneous abortions were noticed and a clear rat infestation problem was observed at the time of the site visit. Serological investigation in sows of this unit at the standard cut-off threshold (1:100) showed low in-herd prevalence. When this cut-off was lowered, the in-herd prevalence was extremely high. Final diagnostic relevance was achieved by confirmation of *Leptospira* antibodies in one aborted litter and the presence of *Leptospira* DNA in a second aborted litter. The low antibody titre suggested that the circulating strain in the swine unit belonged to one of those described of maintenance serovar in pigs. The serogroups mostly found in swine are Pomona, Australis, Tarassovi and Sejroe [14,20,21,22,23], albeit also other serogroups such as Autumnalis, Ballum, Bataviae, Pyrogenes, Grippotyphosa, Icterohaemorrhagiae and Canicola are observed in surveillance studies [21,24,25,26,27]. The predominant serogroup found in the swine unit was Autumnalis, followed by Australis, Tarassovi and Ballum. A 20-year (1988–2007) surveillance study in a reference setting in France shows that the most prevalent serogroup in positive pigs are Icterohaemorrhagiae, followed by Australis and, to a lesser extent, Autumnalis [27]. In the Netherlands, serotypes found between the years 1975 to 1980 in swine were Bratislava, Tarassovi, Ballum and Icterohaemorrhagiae [28]. Despite the different timeframe and variation in predominant serogroups, the result found for this report case are in line with distribution data observed in Belgian neighbouring countries.

Positive PCR results in one litter provided definitive proof of *Leptospira* circulation in this unit. *Leptospira* DNA was detected in all but one piglet at decreasing frequency in the different organs; the spleen in this context was more informative than other organs such as the kidneys and liver, which are classically tested for *Leptospira* tropism. This effect could be associated with maternal transmission of leptospirosis through the placenta and spleen before *Leptospira* transfer to immunologically permissive preferential sites.

Global warming has an important impact on various infectious diseases and this is particularly true of leptospirosis; various reports have put this aspect at the forefront [29,30] and recently human outbreaks worldwide have been associated with flooding [31,32]. Climate conditions were not investigated in this study; therefore, no conclusions could be drawn for this report in relation to climate change.

## 5. Conclusions

In conclusion, despite the fact that there was no direct link between the two animal units, we could demonstrate *Leptospira* circulation within the farm. The use of a lowered cut-off threshold was undoubtedly useful in driving leptospirosis diagnosis. Lowering the MAT cut-off threshold can therefore be justified to support clinical investigations.

## Figures and Tables

**Table 1:** Animal age and sex distribution in the cattle unit.

Age	Male	Female	Total
Younger than 6 months	0	9	9
Between 6 and 12 months	0	2	2
Between 12 and 24 months	0	21	21
Older than 24 months	1	74	75
Total	1	106	107

**Table 2:** Bacteriological and serological results of two swine litters collected between late fall and winter 2012.

Fœtuses	1st Litter		2nd Litter							Ct or Average Ct	Range (Min-Max)
	1	2	1	2	3	4	5	6	7		
Bacteriology											
Kidneys	−	−	+	−	−	−	+	+	−	35.7	34.4–38.0
Liver	−	−	−	−	−	−	+	−	−	38.6	NA
Spleen	nc	nc	+	+	+	+	−	−	−	32.5	30.9–33.4
Lungs	nc	nc	+	−	−	−	−	−	−	38.4	NA
Serology											
MAT 12	1/30 Ballum, Javanica, Australis, Autumnalis, Tarassovi	1/10 Ballum, Javanica, Australis, Autumnalis, Tarassovi	−	−	−	−	−	−	−	NA	

The first litter showed positive MAT12 results in biological fluids, while the second was positive in a bacteriological examination. In light red are highlighted the positive reactions. − indicates a negative result for the test; +: positive; nc: not collected, NA: not applicable.

## Supplementary Materials

The following are available online at www.mdpi.com/2306-7381/4/4/64/s1, Table S1: Panel of *Leptospira* strains used in MAT.
