# Supplementary material for: Reproductive Disorders and Leptospirosis: A Case Study in a Mixed-Species Farm (Cattle and Swine)
Source: Vet Sci. 2017 Dec 1;4(4):64. doi: 10.3390/vetsci4040064 (PMC5753644; doi:10.3390/vetsci4040064)
Supplement: Supplementary file 1 [file vetsci-04-00064-s001.pdf]

veterinary sciences

MDPI

Article

Reproductive Disorders and Leptospirosis: A Case Study in a Mixed-Species Farm (Cattle and Swine)

Marcella Mori [1,*], Raïssa Bakinahe [1], Philippe Vannoorenberghe [1], Jo Maris [2,†], Ellen de Jong [2], Marylène Tignon [3], Martine Marin [1], Damien Desqueper [1], David Fretin [1] and Isabelle Behaeghel [4]

Supplementary Materials

Table S1. Panel of *Leptospira* strains used in MAT.

Species	Serogroup	Serovar	Strain
L. kirschneri	Grippotyphosa	Grippotyphosa	Moskva V
L. interrogans	Canicola	Canicola	Hond Utrecht IV
L. interrogans	Pomona	Pomona	Pomona
L. borgpetersenii	Ballum	Castellonis	Castellon 3
L. interrogans	Icterohaemorrhagiae	Icterohaemorrhagiae	RGA
L. borgpetersenii	Javanica	Poi	Poi
L. interrogans	Australis	Bratislava	Jez Bratislava
L. interrogans	Autumnalis	Autumnalis	Akiyami A
L. interrogans	Bataviae	Bataviae	Swart
L. interrogans	Pyrogenes	Pyrogenes	Salinem
L. borgpetersenii	Tarassovi	Tarassovi	Perepelitsin
L. interrogans	Sejroe	Hardjo type Prajitno	Hardjoprajitno

© 2017 by the authors. Submitted for possible open access publication under the terms and conditions of the Creative Commons Attribution (CC BY) license (http://creativecommons.org/licenses/by/4.0/).
